# Rest-Phase Hypothermia Reveals a Link Between Aging and Oxidative Stress: A Novel Hypothesis

**DOI:** 10.3389/fphys.2020.575060

**Published:** 2020-12-09

**Authors:** Elisavet Zagkle, Marta Grosiak, Ulf Bauchinger, Edyta T. Sadowska

**Affiliations:** ^1^Institute of Environmental Sciences, Faculty of Biology, Jagiellonian University, Krakow, Poland; ^2^Nencki Institute of Experimental Biology, Polish Academy of Sciences, Warsaw, Poland

**Keywords:** rest-phase hypothermia, thermoregulation, aging, body temperature, oxidative stress, food deprivation, birds

## Abstract

In endotherms, growth, reproduction, and survival are highly depended on energy metabolism. Maintenance of constant body temperature can be challenging for endotherms under continuously changing environmental conditions, such as low or high ambient temperatures or limited food. Thus, many birds may drop body temperature below normothermic values during the night, known as rest-phase hypothermia, presumably to decrease energy metabolism. Under the assumption of the positive link between aerobic metabolism and reactive oxygen species, it is reasonable to suggest that low body temperature, a proxy of energy metabolism, will affect oxidative stress of the birds. Aging may considerably affect behavior, performance and physiology in birds and still requires further investigation to understand age-specific changes along the lifespan of the organism. Until today, age-specific rest-phase hypothermic responses and their effect on oxidant-antioxidant status have never been investigated. We exposed 25 zebra finches (*Taeniopygia guttata*) of three age classes, 12 young birds (1.1–1.3 years old), 8 middle-aged (2.4–2.8 years old), and 5 old birds (4.2–7.5 years old) to day-long food deprivation or provided them normal access to food under thermoneutral conditions. We compared night-time body temperature, measured through implanted data loggers, and quantified plasma oxidative status (uric acid, antioxidant capacity, and d-ROM assay) the following morning. We found age-related differences in night-time body temperature following a day-long food deprivation while all three age groups remained normothermic in the night following a day with access to food. The lowest minimum body temperature (LSM ± SE: 36.6 ± 0.2°C) was observed in old individuals during rest-phase hypothermia. Surprisingly, these old birds also revealed the highest levels of plasma oxidative damage, while young and middle-aged birds maintained higher night-time body temperature and showed lower values of oxidative damage. These results lead us to propose a novel hypothesis on how aging may lead to an accumulation of oxidative damage; the impaired physiological capacity to thermoregulate with advancing age does increase the risk of oxidative stress under challenging conditions. When energy is limited, the risk to encounter oxidative stress is increasing via a compensation to defend normothermic body temperatures.

## Introduction

All organisms require energy to grow, reproduce and survive but energy is often a limited resource. Endotherms such as birds and mammals use energy to maintain a relatively constant body temperature over a wide range of ambient temperatures (Scholander et al., 1950). The avian metabolism is remarkably high in comparison to similar-sized mammals (Speakman, 2005; Hulbert et al., 2007) and reflects high endogenous core body temperature in birds between 39 and 44°C (Prinzinger et al., 1991) depending on the physical activity level (resting, low, or high). Such a high metabolism and maintenance of body temperature implies a continual energy consumption and a constant requirement for food supply (Biebach, 1996). However, birds do also encounter a marked variation of environmental conditions, such as cold ambient temperatures or limited food resources, which makes the maintenance of a constant body temperature at normothermic levels a challenge. In consequence, energy-conservation strategies on the level of behavior, physiology, or morphology have evolved to limit the energy use in general, particularly when energy availability is limited.

One such energy-saving strategy is to enter a hypothermic state in which an organism lowers its body temperature and metabolic rate. Three known hypothermic responses are daily torpor, which is common in hummingbirds (Lasiewski, 1963; Bech et al., 1997), hibernation in mammals (Ruf and Geiser, 2015), and rest-phase hypothermia, which is more common in avian species (McKechnie and Lovegrove, 2002; Nord et al., 2009). These three hypothermic responses can be triggered by external stimuli such as limited food availability, although they all differ with respect to their duration and seasonal pattern, their circadian body temperature cycle, and the intensity of the body temperature depression (Reinertsen, 1996). Rest-phase hypothermia is known as a regulated decrease in body temperature by 1–3°C below normothermia during the resting phase (McKechnie and Lovegrove, 2002; Nord et al., 2009) and occurs in diverse mammalian species such as lemurs (Génin and Perret, 2003), rats (Forsum et al., 1981), sheep, and goats (Piccione et al., 2002), and in more than 90 avian species (see review in McKechnie and Lovegrove, 2002). Together with body temperature, metabolic rate decreases considerably during rest-phase hypothermia (Ketterson and King, 1977; Heldmaier and Ruf, 1992; McKechnie and Lovegrove, 2001; Rashotte et al., 2001). White-crown sparrows (*Zonotrichia leucophrys*) decrease metabolic rate by 21% in response to 48 h of food deprivation compared to basal metabolic rate (Ketterson and King, 1977). Metabolic rate decreases by 4–96% from basal metabolic rate, when body temperature drops more than 10°C in 13 avian species (see review in McKechnie and Lovegrove, 2002) and 4–67% similarly in mammals and birds using hibernation or torpor, respectively (Geiser and Ruf, 1995). Such metabolic reduction during hypothermia likely also decreases the rate of enzymatic reactions including those that produce free oxygen radicals (Stefanutti et al., 2005).

Free radicals, including reactive oxygen species (ROS), are regularly formed as a part of aerobic metabolism (Harman, 1956, 1992; Sies, 1997; Rahal et al., 2014) that react with different molecules (Frisard and Ravussin, 2006). When ROS are found in excess and are not quenched, oxidative stress may occur and damage biomolecules such as lipids, proteins or DNA (Davies, 2001). Free radical theory suggests that an accumulation of ROS with increasing age has an inevitable consequence of gradual physiological attrition causing aging (Harman, 1956, 1992; Beckman and Ames, 1998; Finkel and Holbrook, 2000; Höhn et al., 2017). Aging may substantially affect various features such as behavior (Suomi et al., 1996; Verhulst et al., 2014; Labocha et al., 2015), reproductive output (Beamonte-Barrientos et al., 2010), and physiology (Elliott et al., 2015). For instance, a decline in basal or resting metabolic rate with age has been shown in several mammals and birds (see meta-analysis in Elliott et al., 2015), suggesting a decline in body temperature as well. A recent study in birds revealed an age effect on rest-phase hypothermia, old birds (second winter or beyond) had lower night-time body temperature below normothermic values in cold ambient temperature after exposure to predation risk in comparison to the young birds (first experience in winter) (Andreasson et al., 2019). If such an age effect indeed represents physiological attrition with age (senescence) or is rather related to an accumulation of experience to external stimuli remains open and still requires further investigation to understand age-specific changes along the lifespan of the organisms. Age-related differences in night-time body temperature have never been explored for the potential link to the oxidant-antioxidant status in birds.

Rest-phase hypothermia in response to food deprivation may affect the oxidant-antioxidant balance of the organism in different directions. As food deprivation slows down metabolic rate, it may decrease oxidative damage (Sylvie et al., 2012). Food deprivation may also decrease the antioxidant capacity through reduced uptake of exogenous antioxidants (Milinković-Tur et al., 2007). Oxidative stress was lessened in rats after hibernation (Katz et al., 2004; Stefanutti et al., 2005), while in ground squirrels, hibernation induced oxidative stress (Carey et al., 2000). The variation in hypothermic responses and the effect on oxidative status have been previously studied by inter- and intra- specific approaches (Carey et al., 2000; Dede et al., 2002; Alva et al., 2009, 2013), but here we are interested in the age-dependency of the potential link between hypothermic responses and oxidative stress.

We investigated age-specific variation in night-time body temperature and its effect on oxidative stress in zebra finches (*Taeniopygia guttata*) during periods with regular access to food and in response to food deprivation, a manipulation known to elicit rest-phase hypothermia. We evaluated the effect of age by comparing three age groups; twelve young birds (1.1–1.3 years old), eight middle-aged (2.4–2.8 years old), and five old birds (4.2–7.5 years old). Particularly, we predicted that old birds will show the lowest night-time body temperature when provided food under thermoneutral conditions in comparison to the middle-aged and young birds and will exhibit more pronounced hypothermic responses in body temperature in comparison to the middle-aged and young birds when food is deprived. Under the assumption of the positive link between metabolism and ROS production, we further predicted that pronounced rest-phase hypothermia will be associated with oxidative stress.

## Materials and Methods

### Experimental Procedure

Zebra finches (*T. guttata*) are amongst the most common avian model species (Zann, 1996; Griffith and Buchanan, 2010) and a previous study in the same species has shown signs of senescence such as a decline in basal metabolic rate in old individuals (longitudinal study in 1, 3, and 5 years old birds; Moe et al., 2009). Thus, our experimental set up consisted of total 25 birds and we evaluated the effect of age by comparing three age groups, derived from different cohorts originating from our laboratory colony; 12 young birds (1.1–1.3 years old), 8 middle-aged (2.4–2.8 years old), and 5 old birds (4.2–7.5 years old; Supplementary Figure 1). Each age category was represented by even number of males (*n* = 13) and females (*n* = 12), keeping the sex ratio close to 1:1. Males and females were kept separately in similar aviaries (L × W × H: 175 × 140 × 240 cm) in a single climatic chamber under thermoneutrality of 30°C (Calder, 1964) and photoperiodic cycle of 12:12 L:D. A mix of different millet species (Megan, Poland) and freshwater were provided *ad libitum* except during the food deprivation experiment.

At the beginning of the experiment, in the morning just after lights on, we captured all birds to measure body mass using an electronic balance ±0.1 g (KERN 440-45N, Kern & Sohn GmbH, Germany). During the day all birds experienced regular conditions with access to food. In the evening, just before lights off, we measured body mass, removed water, food, and all the seeds from the cage floor. During the experimental period of food and water deprivation, we captured birds in the morning and in the evening for body mass measurements. After 36 h of food and water deprivation, in the morning we captured all birds to obtain body mass and a blood sample for markers of oxidative stress. We allowed birds to recover for 6 days in regular conditions with access to food before we collected a second blood sample in the morning as a control. Before the experimental period, we implemented two short periods (several hours) without access to food and water, to acclimatize birds to irregular food removal, followed by a recovery period of 3 days. We also acclimated the birds to the handling through previously experienced capture and weighing procedure. A scheme of the experimental procedures together with body temperature recordings for each age category throughout the experiment is presented in Supplementary Figure 2.

### Body Temperature Recordings

We assessed the core body temperature of all zebra finches by implanting DST nano-T temperature data loggers (temperature range: 5–45°C; temperature resolution: 0.032°C; accuracy: ±0.2°C; Star-Oddi Ltd., Iceland) in which the recorded body temperature data are stored in the logger’s internal memory (capacity of 43.477 measurements). We synchronized the recordings of the data logger together with a real time clock reference for every 18 min (to compromise between the life battery and the number of saved recordings). The data loggers were 17 mm × 6 mm (length × diameter) and weighed on average 1.3 g which represents 8.1% of the average body mass in zebra finches. We implanted the data loggers inside the intraperitoneal cavity under inhalation anesthesia with 3% of isoflurane (Aerrane Isofluranum, Baxter, Belgium) at 0.3 L/min of airflow. During anesthesia, the birds were placed with their bill inside a closed-pipe inhalation system (UNO200VAP vaporizer, Scintica Instrumentation inc., Maastricht, Netherlands). Before surgery, data loggers were disinfected with 95% ethanol and lightly coated with a lubricant (vaseline 80% and paraffin wax 20%). We gently slid in the abdominal cavity the data loggers via a midline incision (about 0.5–1 cm). Two to three stitches (Safil 5/0, AesculapAG, Tuttlingen, Germany) were employed for the closure of the skin opening. Surgery room had a constant room temperature (21°C) and birds were placed on a heat mat to keep them warm during the anesthesia. After implantation, we let birds recover for 4 days and monitored their condition by measuring their body mass and observing the wound. The logger implantation took place 6 weeks before the food deprivation. After this experiment, birds were assigned to a different study and at the end, they were dissected for organ mass and lean mass. We removed the loggers and we downloaded the body temperature recordings using the SeaStar program (Star-Oddi Ltd., Iceland). Data loggers were calibrated using a water bath and a thermometer (accuracy: ±0.4°C; WD-35427-52 Temp 300, Oakton Instruments, IL, United States) in four calibration temperatures from 30 to 45°C.

### Oxidative Status Biomarkers

Enzymatic and non-enzymatic antioxidants play a crucial role as scavengers of ROS components and may mitigate the negative effects of oxidative stress. In birds, it has been suggested that a potent endogenously produced antioxidant is uric acid (Tsahar et al., 2006). Birds excrete mostly uric acid as a metabolized nitrogen end-product and thus plasma uric-acid concentration may be used as an index of protein catabolism (Boismenu et al., 1992; Cohen et al., 2008). To estimate oxidative status of the birds, we measured concentrations of oxidative damage, non-enzymatic antioxidant capacity and uric acid as a potent antioxidant in plasma using the d-ROM, the oxy-adsorbent (OXY) and uricase colorimetric tests respectively (Diacron International, Grosseto, Italy). We captured all birds in the morning just after lights on, at the end of food deprivation conditions, and collected blood samples. After 6 days of recovery under *ad libitum* food, we obtained a second blood sample during the same time of the day. Blood samples were obtained from the wing vein of the birds, collected in heparinized capillaries and immediately centrifuged for 10 min at 3340 *g* to separate plasma (Centrifuge MPW-56, MPW Med. Instruments). Plasma was stored at −80°C until further analyses. Following the protocol of Costantini and Dell’Omo (2006) for the d-ROM test, we estimated oxidative damage and the results are expressed as mmol of H_2_O_2_ equivalents. The OXY test measures the total non-enzymatic antioxidant capacity (Costantini and Dell’Omo, 2006) by quantifying the ability of plasmatic antioxidants to cope with hypochlorous acid (HClO); an endogenously produced oxidant. We followed the protocol of Sudyka et al. (2016) and values are expressed as mmol of HClO neutralized. The plasma concentration of uric acid was measured by the endpoint uric acid assay kit (Diacron International, Grosseto, Italy). In each well of a 96-well microplate, 2.5 μl of plasma (similarly for blank sample and standard) was mixed with 100 μl of reagent, incubated and shaken in 37°C for 5 min and measured in 510 nm wavelength. The colorimetric uric acid test uses uricase enzyme to convert uric acid to allantoin and the reaction with hydrogen peroxides allows the spectrophotometer to read the concentration of uric acid. The values of uric acid in the plasma are expressed as mmol/L. All analyses for the colorimetric assays were run in duplicate using an absorbance reader (Sunrise, Tecan’s Magellan, Tecan Trading, Switzerland). In the end, we estimated oxidative stress index by calculating the ratio of the oxidative damage to the total non-enzymatic antioxidant capacity in the plasma multiplied by 1000.

### Statistical Analysis

We present the analysis and results on body temperature and oxidative biomarkers, while all the analysis and results on body mass and body mass loss are presented as Supplementary Material. We calculated the mean (*T_*b mean*_*) and minimal (*T_*b min*_*) body temperature for each individual. We further calculated median (*T_*b median*_*) and maximum (*T_*b max*_*) body temperature, with these results reported in the Supplementary Material. We performed analysis of covariance to test the effect of age and the photoperiod in body temperature, with age and photoperiod of the chamber as categorical variables and body mass as a covariate. The day in the chamber was defined from the moment of lights on (8:00 a.m.) until lights off (8:00 p.m.). Due to significant differences in body temperature between day and night (Supplementary Table 1), we further performed statistical analysis separately for day and night. We performed an analysis of covariance to test whether the age of the birds is related to night-time *T_*b mean*_* or *T*_*b min*_ after a day with access to food and a separate analysis for the night-time *T_*b mean*_* or *T_*b min*_* after long-day food deprivation. The age group (young, middle-aged, and old birds) was included as a categorical factor and the body mass was included as a covariate variable.

We ran a linear mixed-effect model to account for the repeated measurements for night-time *T_*b mean*_* and *T_*b min*_* to test age-related differences in response to a day with access to food and without food. The effect of age group (young, middle-aged, old birds), food manipulation (with food, without food) and the interaction between them were included in the model as fixed factors. Individual identification number was included as a random effect to account for the repeat measurements. The oxidative stress biomarkers were analyzed similarly as body temperature data. We performed all statistical analysis using R Studio software (R version 3.5.3, R Core Team, 2019). All variables verified the assumption of normality and homoscedasticity, except of the oxidative stress index, and thus it was log-transformed. Linear mixed effect models were performed using “lmer” function from lme4 package (Bates et al., 2015). Estimated means were obtained using the emmeans package (Lenth, 2018).

## Results

### Body Temperature

**FIGURE 1:**
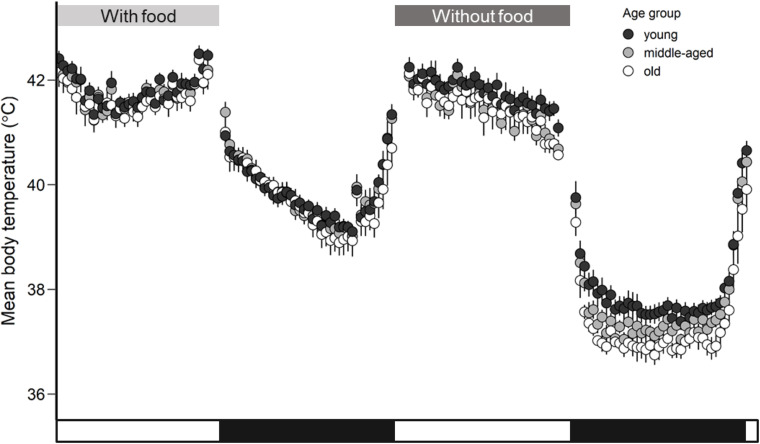
Mean body temperature (°C) calculated for each age group (young, middle-aged, old) of zebra finches with access to food and rest-phase hypothermia in response to a day without access to food. Whiskers represent standard errors. Black points represent the young group, light gray the middle-aged and white the old group of birds. The white and black bars in the *x*-axis correspond to the light and dark phase.

Body temperature showed a circadian rhythm when food and water were provided *ad libitum* in thermoneutral conditions for all birds (Figure 1). Zebra finches demonstrated a *T_*b mean*_* of 41.8 ± 0.1°C (LSM ± SE) during the daytime and they exhibited a drop by 2.1°C during the nighttime to 39.7 ± 0.1°C (*F*_1_,_41_ = 255.29, *p* < 0.001). Because of the statistical significance of the day-night effect (Supplementary Table 1), we further analyzed *T_*b mean*_* and *T_*b min*_* separately for day and night. When birds had access to food the *T_*b mean*_* did not differ among age groups during daytime (*F*_2_,_20_ = 0.35, *p* = 0.71) and during nighttime (*F*_2_,_20_ = 0.15, *p* = 0.86). Similarly, the age factor was not found significant for *T_*b min*_* during daytime (*F*_2_,_20_ = 0.14, *p* = 0.87) and during nighttime (*F*_2_,_20_ = 0.35, *p* = 0.71). The *T_*b mean*_* was not related to body mass (*p* = 0.31) and neither was the *T*_*b min*_ (*p* = 0.39). The estimated means and significance of the effects from the analysis of covariance for *T*_*b mean*_, *T*_*b min*_, *T*_*b max*_, and *T*_*b median*_ are shown in Supplementary Table 2.

All birds significantly reduced night-time body temperature in comparison to day-time body temperature by an average of 4.2°C in response to the food deprivation conditions. Such a drop of night-time body temperature below normothermic values is characterized as rest-phase hypothermia (McKechnie and Lovegrove, 2002). The drop in *T_*b mean*_* during rest-phase hypothermia differed by about 2.1°C (*F*_1_,_117_ = 915.97, *p* < 0.001) in comparison to the *T_*b mean*_* during normothermic night (Figure 1). The estimated means and significance of the effects from the analysis of covariance for *T*_*b mean*_, *T*_*b min*_, *T*_*b max*_, and *T*_*b median*_ are shown in Table 1. The performed analysis of covariance showed that the *T_*b mean*_* during the day was not related to age factor (*F*_2_,_20_ = 1.01, *p* = 0.38) but interestingly, the *T_*b mean*_* during the night was significantly related to the age factor (*F*_2_,_20_ = 4.46, *p* = 0.02) in response to food deprivation conditions. The significant interaction in linear mixed effect analysis between age and food manipulation shows that the age effect in *T_*b mean*_* depends on the food manipulation with old birds exhibiting lower *T_*b mean*_* (LSM ± SE: 37.9 ± 0.1°C) than the young birds (LSM ± SE: 37.3 ± 0.2°C) during rest-phase hypothermia.

**TABLE 1:** Results of analysis of covariance (ANCOVA) of T_*bmean*_, T_*bmin*_, T_*bmax*_, and T_*bmedian*_ (°C) in response to food deprivation conditions separately for day and night.

Variable	Young		Middle-aged		Old		Age factor			Body mass		
	LSM	SE	LSM	SE	LSM	SE	*F*	df	*p*	*F*	df	*p*
Day												
*T_*b*_ mean*	41.8	0.1	41.4	0.2	41.5	0.2	1.01	2,20	0.38	1.28	2,20	0.27
*T_*b*_ min*	40.8	0.1	40.5	0.2	40.4	0.2	1.41	2,20	0.27	0.03	2,20	0.87
*T_*b*_ max*	42.6	0.0	42.3	0.1	42.5	0.1	1.44	2,20	0.26	6.38	2,20	**0.02**
*T_*b*_ median*	41.8	0.1	41.4	0.2	41.5	0.2	0.94	2,20	0.41	1.18	2,20	0.29
Night												
*T_*b*_ mean*	38.0	0.1	37.5	0.1	37.2	0.2	4.46	2,20	**0.02**	0.31	2,20	0.58
*T_*b*_ min*	37.2	0.1	36.7	0.2	36.6	0.2	3.40	2,20	**0.05**	0.24	2,20	0.63
*T_*b*_ max*	40.6	0.2	40.1	0.2	39.9	0.3	2.50	2,20	0.11	2.32	2,20	0.14
*T_*b*_ median*	37.7	0.1	37.3	0.2	37.0	0.2	4.37	2,20	**0.02**	0.24	2,20	0.63

*Age group, 12 young (1.1–1.3 years old), 8 middle-aged (2.4–2.8 years old), and 5 old (4.2–7.5 years) birds, was included as a categorical variable. Body mass (g) was recorded just after lights on and was included as a covariate in the model. Least squares means (LSM), standard error (SE), statistics; *F*-value, degrees of freedom for numerator and denominator (df) and *p*-value based on Satterthwaite’s method approximation. Bold letters indicate statistical significance of the effect smaller or equals to 0.05.*

**FIGURE 2:**
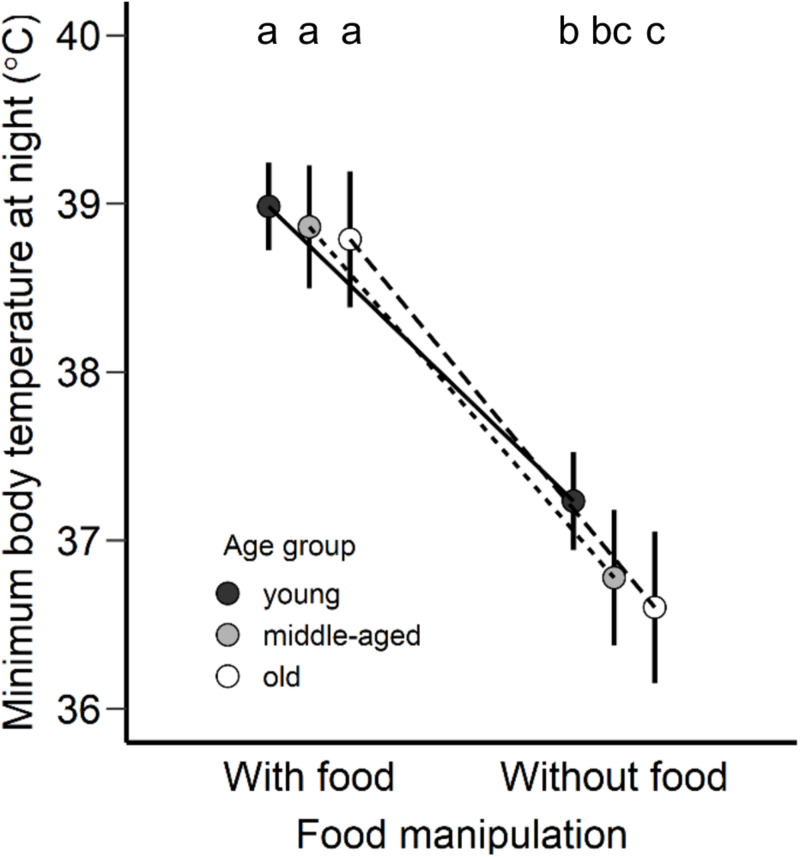
Minimum body temperature at night (°C) adjusted as least square means for each age group (young, middle-aged, old) of zebra finches with access to food and without food. Points are presented as least square means and error bars represent the 95% confidence intervals. Different letters represent statistical differences after *post hoc* Tukey’s comparison.

In the case of *T_*b min*_* during rest-phase hypothermia, all birds were characterized by a lower *T_*b min*_* by about 4.4°C in comparison to the *T_*b min*_* during the day in response to food deprivation conditions. The age was not found to be a significant factor during the daytime (*F*_2_,_20_ = 1.41, *p* = 0.27), albeit was found pronounced during the night (*F*_2_,_20_ = 3.40, *p* = 0.05). Old birds revealed lower *T_*b min*_* (LSM ± SE: 36.6 ± 0.2°C) than the young birds (LSM ± SE: 37.2 ± 0.1°C) during rest-phase hypothermia (Figure 2, *post hoc* Tukey’s comparison).

### Oxidant– Antioxidant Status

**FIGURE 3:**
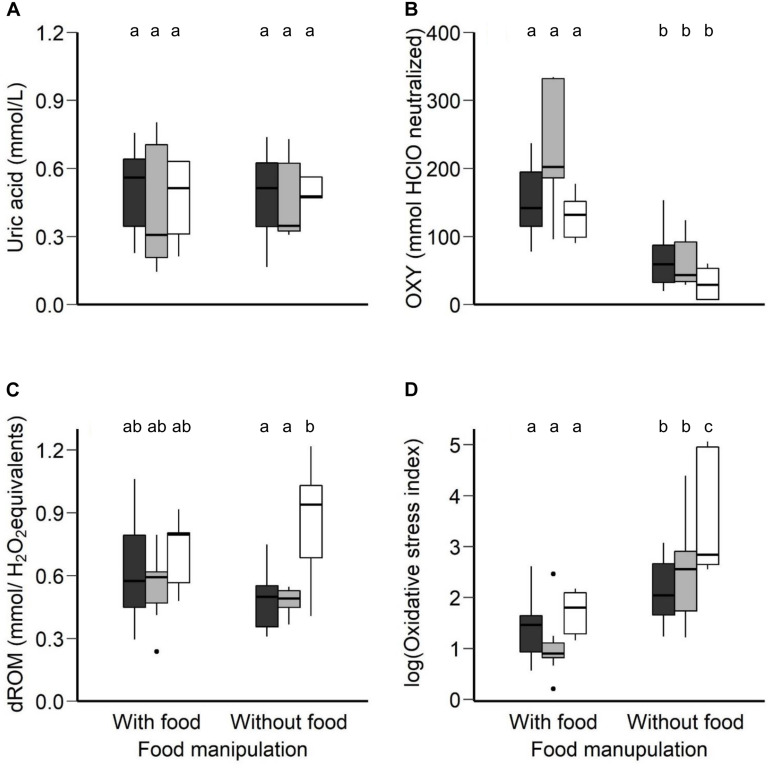
**(A)** Uric acid (mmol/L), **(B)** OXY (mmol HClO neutralized), a metric for non-enzymatic antioxidant capacity, **(C)** dROM (mmol/H_2_O_2_ equivalents), a metric of oxidative damage, in plasma concentration and **(D)** oxidative stress index (log-transformed), calculated as the ratio of the oxidative damage to the total non-enzymatic antioxidant capacity in the plasma multiplied by 1000, in the morning following normal night-time body temperature with access to food and rest-phase hypothermia without access to food between the three age groups (young, middle-aged, old) of zebra finches. Boxplots show the median, the range and the inter-quartiles. Black boxplots represent the young group, light-gray the middle-aged group and the white the old group of birds. Different letters represent statistical differences after *post-hoc* Tukey’s comparison.

The uric acid in plasma did not vary between the food manipulation conditions (*F*_1_,_18_._9_ = 0.05, *p* = 0.82) and between the three age groups (*F*_2_,_19_._9_ = 0.41, *p* = 0.66). The interaction was not significant (*F*_2_,_18_._9_ = 0.02, *p* = 0.97; Figure 3A).

The non-enzymatic antioxidant capacity in plasma significantly differed between the two food manipulation conditions (*F*_1_,_39_ = 38.13, *p* < 0.001; Figure 3B), but not between the age groups (*F*_2_,_39_ = 1.27, *p* = 0.29; Figure 3B). The non-enzymatic antioxidant capacity was significantly higher after a regular night-time body temperature with access to food (LSM ± SE: 165.83 ± 13.10 mmol HClO neutralized) than after rest-phase hypothermia in response to a day-long food deprivation (LSM ± SE: 51.70 ± 13.10 mmol HClO neutralized, *post hoc* Tukey comparison: *t* = −6.16, *p* < 0.001). The interaction between food manipulation and the age effect was not significant (*F*_2_,_39_ = 1.75, *p* = 0.19).

The oxidative damage in plasma did not differ in response to the different food manipulation conditions (*F*_1_,_21_._25_ = 0.20, *p* = 0.66; Figure 3C), but did differ between age groups (*F*_2_,_21_._84_ = 5.79, *p* = 0.01; Figure 3C). The interaction of age group and food manipulation was found to have a trend and thus we performed analysis of covariance separately for each condition; food availability and food deprivation (body mass as a covariate). The morning after normothermic night in response to a day with access to food, the oxidative damage in plasma did not differ between age groups (*F*_2_,_19_ = 1.01, *p* = 0.38). To the contrary, for the morning after rest-phase hypothermia in response to a day without access to food, we found age-related differences (*F*_2_,_21_ = 9.93, *p* < 0.001). Old birds revealed higher oxidative damage than middle-aged (*t* = −3.24, *p* = 0.01) and young birds (*t* = −4.11, *p* = 0.001) after *post hoc* Tukey comparison.

Oxidative stress index, calculated as the ratio of oxidative damage to the total non-enzymatic antioxidant capacity in the plasma multiplied by 1000 (Costantini et al., 2007), after log-transformation was found to differ significantly between the food manipulations (*F*_1_,_37_ = 38.05, *p* < 0.001; Figure 3D) and between the age groups (*F*_2_,_37_ = 6.18, *p* = 0.004; Figure 3D). The interaction between age group and food manipulation may be interpreted as significant trend (*F*_2_,_37_ = 2.74, *p* = 0.07). The age effect in oxidative stress index depends on the food manipulation with old birds revealing higher oxidative stress than the young birds in response to a day-long of food deprivation and rest-phase hypothermia (Figure 3D; *post hoc* Tukey comparison, *t* = −3.91, *p* = 0.001). The estimated means and significance of the effects from the linear mixed effect analysis for all the oxidative and antioxidant status biomarkers measured in our study to test age-related differences between the different food manipulation groups are presented in Supplementary Table 3.

## Discussion

This study shows age-specific differences in body temperature during rest-phase hypothermia and different levels of oxidative damage in the morning; older zebra finches exhibited stronger hypothermic responses (Figures 1, 2) and revealed higher levels of oxidative stress compared to younger birds (Figure 3). Both the core body temperature and the level of oxidative stress were different between age groups after one day without access to food, while such differences were not detectable after a day with access to food. Our data are in agreement with the concept of a progressive accumulation of damage with increasing age (Beckman and Ames, 1998; Finkel and Holbrook, 2000; Weinert and Timiras, 2003; Höhn et al., 2017) and in our study only occurred in conjunction with the environmental stressor of food deprivation. Thus, we promote a new hypothesis for the causality of the increased oxidative stress at higher age through age-specific changes in thermoregulation.

It appears unlikely that the here observed age-related differences in body temperature during rest-phase hypothermia could be due to behavioral responses that differ between the age groups. Birds may employ huddling to minimize thermoregulatory costs during cold ambient temperatures (see Gilbert et al., 2010) and thus save energy (Burns et al., 2013). Age is also known to alter hierarchies in social groups and affect many kinds of behavior (Verhulst et al., 2014). However, in our study, we maintained birds under thermoneutral conditions, and thus we removed all thermoregulatory costs including the benefit to save energy through huddling. Differences in body temperature and oxidative stress are rather a consequence of physiological differences between the different age groups instead of behavioral responses, and we consider it likely that body temperature (Figure 1) in fact drive the result of elevated oxidative stress in older birds compared to younger ones (Figures 3C,D).

Birds, during rest-phase hypothermia, lower body temperature and metabolic rate as an energy-saving strategy (McKechnie and Lovegrove, 2002), but here the lack of age-related differences in body mass change (Supplementary Figure 3B) does not seem to support an age-specific energy-saving hypothesis. The stronger hypothermic responses of older-aged zebra finches compared to younger conspecifics entail fundamental differences between the different aged birds that are tightly linked to energy metabolism in general. In particular, (i) on average lower body temperature over the whole night results in accumulation of a lower rate of metabolism, (ii) lower minimum body temperature is also linked to a lower rate of metabolism but rather to a single moment of low metabolism that can be even more pronounced than average nighttime body temperature, and (iii) lower body temperature during the night entails higher energetic demands during rewarming until normothermic levels are reestablished.

An imbalance between pro-oxidants and antioxidants toward the oxidants may lead to oxidative stress (Sies, 1997), and result in damage of various biomolecules that may impair their proper functioning (Gutteridge and Halliwell, 2000). Old zebra finches in this study showed significantly higher oxidative damage compared to younger ones after a night with rest-phase hypothermia (Figure 3C) in response to food deprivation. However, the question of if and how the age-specific differences in body temperature may relate to the documented oxidative damage remains unclear. We consider three major steps in the oxidative stress cascade that may drive the high oxidative damage with increasing age, and these three steps may even follow a hierarchy as they can be applied at different stages in the process to maintain the functioning of biomolecules. Initially, the body of an organism responds with the first line of defense, by avoiding the excessive production of free radicals (Sies, 1997; Young and Woodside, 2001). Secondly, once ROS are formed, the organism may scavenge them. Thirdly, if ROS production could not be avoided or detoxified and indeed biomolecules encountered oxidative damage, such damaged biomolecules could be repaired or even entirely replaced or removed (Halliwell, 1994; Young and Woodside, 2001; Rahal et al., 2014). In the following section, we will discuss how our results on age-specific body temperature during rest-phase hypothermia may explain the increased oxidative damage of old birds through potentially differential actions on processes of the cascade from energy metabolism to oxidative damage.

### Mean Night-Time Body Temperature Indicative for Continuous Differences

Old zebra finches had, on average, lower body temperature throughout the entire night compared to younger ones, suggesting a considerable effect on metabolic rate. Zebra finches decrease night-time metabolic rate by 55% after day-long food deprivation (14 h) in comparison to a normal night (Rashotte et al., 2001). Such a drop in metabolic rate should also decrease ROS production, and either lower oxidative stress or reduce the need for countermeasures against ROS. Oxidative damage in mallards was reduced by 95% after 2 days of food deprivation compared to feeding conditions (Sylvie et al., 2012). While food deprivation may decrease ROS production, presumably via a reduction in metabolism (Sylvie et al., 2012), it may also decrease the exogenous antioxidants via food intake (Milinković-Tur et al., 2007). However, the published results for the antioxidant status are ambiguous, with lowered levels in cockerels after 2 days of fasting in comparison to control with access to food conditions, while antioxidant status remained unchanged in pullets (Milinković-Tur et al., 2007). In the mallard study, after two fasting days, non-enzymatic antioxidant capacity remained unchanged (Sylvie et al., 2012). Non-enzymatic antioxidant capacity measured in our study was significantly decreased after rest-phase hypothermia following food deprivation when compared to normal access to food (Figure 3B), but it did not differ between the age groups. However, the level of oxidative damage following food deprivation is lower in young and middle-aged birds when compared to regular conditions with access to food, while it is higher in old birds (Figure 3C). Contrary to our prediction, old birds with lower average body temperature during the night and hence a presumed lower metabolic rate, revealed higher oxidative damage compared to younger birds.

Instead of the positive link between metabolism and ROS production, the decrease in body temperature might itself explain the differences in oxidative damage levels between the age groups, since enzyme functioning and the relative antioxidant activity are highly affected by temperature (Hove and Hove, 1944; Geiser and McMurchie, 1985; Elias et al., 2014). Enzyme functioning and the antioxidant activity may be different between the age classes as old birds showed the most pronounced drop in body temperature during rest-phase hypothermia compared to more moderate responses of young and middle-aged birds. A study on mice revealed an increased ROS production in isolated mitochondria in low temperatures of 35 and 32°C, which resemble body temperature during hypothermic responses, in comparison to 37°C (normothermia) (Ali et al., 2010). As mitochondria are the main source for ROS production (Sohal et al., 1994; Speakman et al., 2004) the increased oxidative damage in old birds may be due to the higher ROS production at the mitochondria level compared to that of young birds. It is possible that changes in the respiratory chain activities and low body temperatures may affect mitochondrial efficiency and proton leak (Sylvie et al., 2012). A study in ectotherms showed that lower metabolic rate and food deprivation resulted in increased mitochondrial coupling efficiency by lowering the proton leak in liver mitochondria (Trzcionka et al., 2008, but see in heterotherms Polymeropoulos et al., 2017). We may only speculate that old individuals of our study increased mitochondrial coupling efficiency by lowering proton leak at the costs of higher oxidative damage. The low body temperature of the oldest cohort of zebra finches could significantly influence the production of ROS levels or/and the antioxidant enzymatic activity which could scavenge the high levels of ROS. Antioxidant enzymatic activity in erythrocytes was decreased in rats after 1 h in induced hypothermia via a cooling table (Alva et al., 2013), and under cold stress conditions (15 min/per day in 5°C) (Gümüşlü et al., 2002). During short-term hypothermia, lipid peroxidation increased and the GSH (glutathione, an antioxidant enzyme) levels were decreased in rats (Dede et al., 2002). Clearly, antioxidant activity or oxidative stress are affected by modulation of body temperature beyond the normothermic levels. Our data show overall night-time body temperature is the lowest in older birds and may be linked to oxidative stress, but only under food deprivation conditions, while during steady-state conditions with regular access to food, neither a response to body temperature or oxidative stress was elicited in any age groups.

### Minimum Night-Time Body Temperature Indicative for Maximum Differences

**FIGURE 4:**
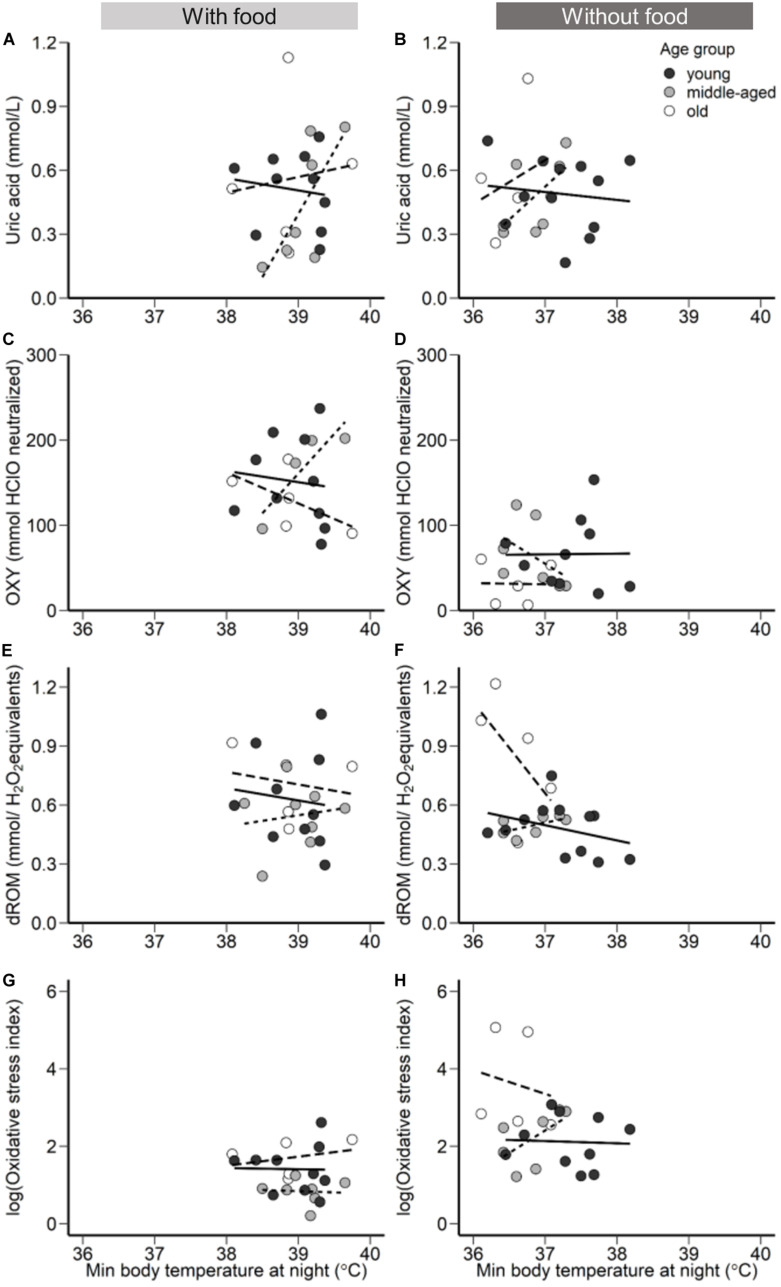
The relationship between minimum body temperature during the night with access to food (left panels) and without access to food (right panels). **(A,B)** Uric acid (mmol/L), **(C,D)**, OXY (mmol HClO neutralized), a metric for non-enzymatic antioxidant capacity, **(E,F)** dROM (mmol/H_2_O_2_ equivalents), a metric of oxidative damage, in plasma concentration, and **(G,H)** Oxidative stress index (log transformed), calculated as the ratio of the oxidative damage to the total non-enzymatic antioxidant capacity in the plasma multiplied by 1000. Points represent minimum body temperature values for each individual during the night following a day with access to food (left panels) and following a day without access to food (right panels). Black filled circles and solid regression line represent young group, gray filled circles and dotted regression line the middle-aged, and white filled circles and the dashed regression line old group of birds.

The effects of hypothermia on the pro-oxidant-antioxidant system can differ depending on the duration and the drop of the body temperature (Alva et al., 2013). Rest-phase hypothermia is characterized by an overall metabolic reduction and maintenance of low body temperature throughout the entire night, which at a specific time point reaches a minimum body temperature set point (McKechnie and Lovegrove, 2002). Thus, the above outlined effect of low temperature on energy metabolism or enzyme activity may accumulate over a longer period (see the previous section), or the momentary minimum body temperature has an immediate but lasting effect. In this study, minimum body temperature revealed age-related differences (Figure 2) similar to the effect of mean body temperature in response to a day-long without access to food, and both minimum and mean body temperature appear to be associated to oxidative status (Figure 4 and Supplementary Figure 4). This relationship indicates an age-dependency in body temperature only under environmental challenges when maintenance of body temperature seems to be challenging, however further research and experimentation is required.

### Metabolic Demands During Rewarming

While rest-phase hypothermia decreases metabolic rate and, presumably, also enzymatic reactions, the phase of re-warming the body back to normothermic levels at the end of the night may reverse all these metabolic effects. Re-warming takes place toward the end of the night following rest-phase hypothermia (Geiser and Ruf, 1995; Reinertsen, 1996), and considering our data, higher energetic demands must be required for the old birds to re-warm compared to younger ones. Our estimation of the re-warming costs (based on the formula from McKechnie and Wolf, 2004) did not yield such age-related differences (Supplementary Figure 5). Given the complexity of the true re-warming costs and the crude estimation approach we used here, we think that direct respirometric and body temperature measurements of rewarming in the context of effects on oxidative status offer a promising avenue for increased understanding of how body temperature may mediate age-associated oxidative stress.

### Impaired Thermoregulation With Increasing Age as a Potential Driver of Damage Accumulation

Aging seems to have considerable effects on the thermoregulatory capacity and can impair specific mechanisms for maintenance of body temperature under normothermic values (Refinetti et al., 1990; Van Someren et al., 2002; Flouris and Piantoni, 2015). For instance, basal metabolic rate seems to decline with increasing age in birds and mammals (see meta-analysis in Elliott et al., 2015), which may hint to a lower body temperature. Lower body temperature with increasing age has been shown in several studies (see review Kelly, 2006; Andreasson et al., 2020) and such a decrease in body temperature with age may be related to the inability to produce more heat or maintain the heat. For instance, changes in body fat with advancing age might explain lower body temperature in elderly humans in comparison to young (Van Someren et al., 2002; Kenney and Munce, 2003; DeGroot and Kenney, 2007), while in birds subcutaneous fat-loss could affect insulation and the capacity to maintain heat. Reduced peripheral vasoconstriction or increased peripheral vasodilation might also affect the ability in old organisms to lose more heat (Van Someren et al., 2002; Holowatz and Kenney, 2010). Differences in the organisms’ thermal sensation with progressive age may also explain the observed age-related decline of thermoregulatory functions (Xiong et al., 2019). A study on mammals has shown that the age-related decline in cutaneous vasoconstrictor responses to cold exposure is due to a weaker signal from the periphery to the regulatory centers (Bicego et al., 2007). Such effects in one or multiple various functions in endotherms’ thermoregulatory capacity with increasing age should modulate basal metabolic rate and it would be interesting for a future study to implement direct respirometric measurements to evaluate any compensation in basal metabolic rate.

In response to food deprivation, old birds lowered body temperature more than young ones suggesting an impaired ability to either produce or conserve heat. Therefore, we forward a novel hypothesis stating that age-specific increase in oxidative stress is mediated by impaired thermoregulatory capacity. Hereby, the increased challenge to maintain body temperature within a normothermic range may affect multiple metabolic levels ranging from temperature-dependent enzyme activity over the link of the energy metabolism to body temperature to the need to restore normothermic levels following fluctuations of body temperature, all of which may affect single or multiple levels of the oxidative-antioxidant cascade. What precisely causes animals to lose the thermoregulatory capacity with increasing age remains to be unveiled, but our hypothesis promotes that the further an animal leaves normothermic levels, the higher is the risk for oxidative damage (Figure 4).

“Aging is defined as the time-dependent persistent change of functionality and reproducibility, affecting all higher organisms” (Höhn et al., 2017). Many aging theories (more than 300, reviewed by Medvedev, 1990) have been forwarded to explain what causes such a change in functionality (Medvedev, 1990; Beckman and Ames, 1998; Weinert and Timiras, 2003; Monaghan et al., 2008), and at present theories that predict an increase in oxidative stress through either accumulation of ROS, the decline in defense against ROS, or the accumulation of oxidative damage appear the most promising ones. Here we formulate a new hypothesis for what may cause such an increase in oxidative stress with progressing age, namely the age-dependent decline in thermoregulatory capacity. Our current data promotes that such a decline in thermoregulatory capacity may translate into oxidative stress only under particularly challenging conditions and not in day-to-day life. This new hypothesis advocates to direct research activities to the change in thermoregulation and how an organisms’ thermoregulatory capacity may decrease with age if we want to better understand the overall aging process.

## Data Availability Statement

The original contributions presented in the study are included in the article/Supplementary Material, further inquiries can be directed to the corresponding author/s.

## Ethics Statement

The animal study was reviewed and approved by II Local Bioethical Committee in Kraków, Poland.

## Author Contributions

UB, ES, and EZ conceived the experiment. EZ performed the experiment, carried out all the measurements, and analyzed the data with the help of MG and ES. EZ and UB wrote the manuscript. All authors commented on the manuscript.

## Conflict of Interest

The authors declare that the research was conducted in the absence of any commercial or financial relationships that could be construed as a potential conflict of interest.
