# Supplementary material for: Rest-Phase Hypothermia Reveals a Link Between Aging and Oxidative Stress: A Novel Hypothesis
Source: Front Physiol. 2020 Dec 9;11:575060. doi: 10.3389/fphys.2020.575060 (PMC7756103; doi:10.3389/fphys.2020.575060)
Supplement: Supplementary file 1 [file Table_1.docx]

SUPPLEMENTARY MATERIAL

**Rest-phase hypothermia reveals a link between aging and oxidative stress: a novel hypothesis**

Elisavet Zagkle^1*^, Marta Grosiak^1^, Ulf Bauchinger^1,2^, Edyta T. Sadowska^1^

^1^ Institute of Environmental Sciences, Faculty of Biology, Jagiellonian University, Krakow, Poland

^2^ Nencki Institute of Experimental Biology, Polish Academy of Sciences, Warsaw, Poland

*** Correspondence:**Corresponding Author
elisavet.zagkle@doctoral.uj.edu.pl

Number of supplementary figures: 5

Number of supplementary tables: 3


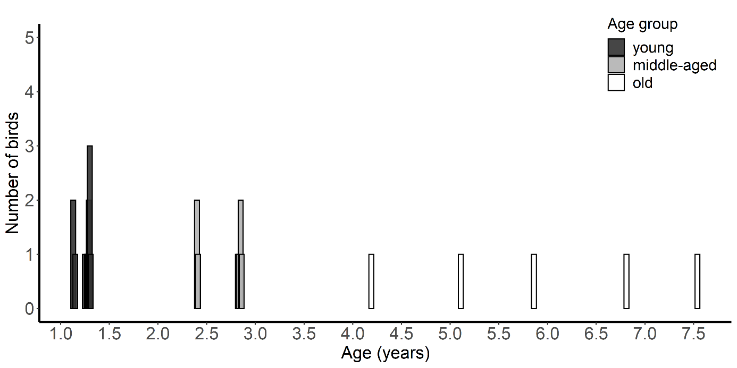


**Supplementary Figure 1|** Age (years) distribution of the experimental set up of birds. Black bars represent the young group (n = 12), light grey the middle-aged (n = 8) and white the old group (n = 5) of birds.

*
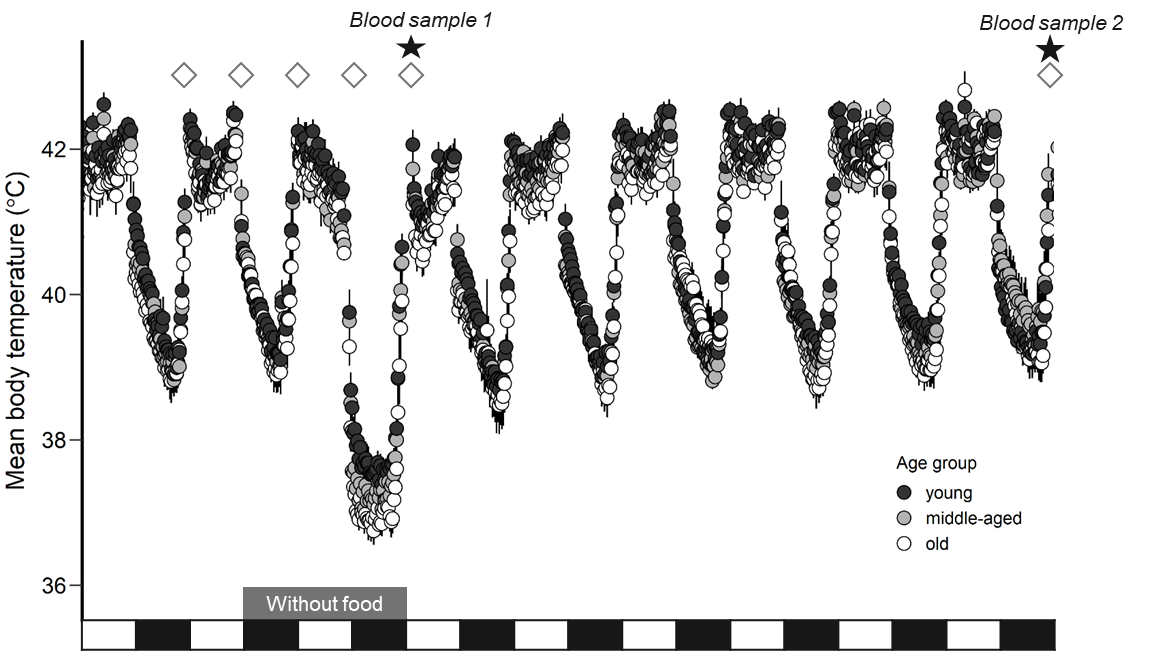
*

**Supplementary Figure 2|** Mean body temperature (°C) calculated for each age group (young, middle-aged, old) of zebra finches along the experimental days. Whiskers represent error bars. Black circles represent the young group, light grey the middle-aged and white the old group of birds. The white and black bars in the x-axis correspond to the light and dark phase. Body mass measurements are represented with the diamonds and the blood sampling points with the stars.

1. **Body mass and body mass change**

We measured body mass in the morning immediately after lights on and evening just before lights switch off using an electronic balance ±0.1 g (KERN 440-45N, Kern & Sohn GmbH, Germany). To test age-related differences in body mass we performed one-way ANOVA. To test if birds maintained their body mass from one morning until the following morning when they had access to food and without access, we performed linear mixed effect model. Age and morning time points of measurements were included and the interaction between them as fixed effects. Individual identity was included in the model as a random effect. Additionally, we calculated body mass change over 12 hours during the day of food deprivation (from morning to evening), over 12 hours during the night after long-day food deprivation (from the evening to the following morning) and last, over 24 hours (from one morning to the following morning). Body mass change was set as a response variable in the analysis of covariance to test age-related differences in body mass change over the day, night and over 24 hours during food deprivation conditions. Initial body mass of each bird was included as a covariate.

Body mass at the beginning of the experiment (initial body mass) measured immediately after lights on did not differ among the age groups (mean ± SE: 16.1 ± 0.09, F_2,21_ = 3.18, p = 0.06)*.* Similarly, body mass after lights on following food deprivation conditions did not differ among the age groups (mean ± SE: 14.6 ± 0.32, F_2,21_ = 2.51, p = 0.11)*.* Body mass of the birds for the three age groups measured immediately after lights on (morning) and just before lights off (evening) is depicted during the day with access to food and during the day without access to food in Supplementary Figure 3A. Repeated measured analysis showed that all birds maintained their body mass from one morning to the following morning (F_1,21_ = 0.35, p = 0.56) when food was available, without age-related differences (Supplementary Figure 3A). All birds significantly decreased their body mass by 1.8 g (F_1,21_ = 2367.52, p <0.001) from one morning (LSE ± SE: 16.3 ± 0.3) to the following morning (LSE ± SE: 14.5 ± 0.3) during food deprivation, without age-related differences (Supplementary Figure 3A).

When food was available, analysis of covariance showed that body mass change during the day did not differ among age group (F_2,21_ = 0.68, p = 0.52, Supplementary Figure 3B) while body mass change during the night showed a tendency for age-related differences (F_2,20_ = 3.29, p = 0.05, Supplementary Figure 3B). Old birds lost more body mass (LSE ± SE: 1.5 ± 0.1 g) than the young birds (LSE ± SE: 1.3 ± 0.1 g) during the night following a day with access to food (post-hoc Tukey comparison, t = 2.32, p = 0.07).

During food deprivation, body mass change over the day differed between age group (F_2,20_= 4.98, p = 0.01). This was affected from one individual but when removing the individual the significance of the age effect remained (F_2,19_ = 5.68, p = 0.01). Middle-aged birds lost more body mass than the young birds (post-hoc Tukey comparison, t = -1.98, p = 0.14). Body mass change was significantly related to body mass over the day (p < 0.05). Body mass change over the night did not differ between the age groups (F_2,20_ = 0,39, p = 0.68) and neither body mass was related to body mass over the night (p = 0.13).


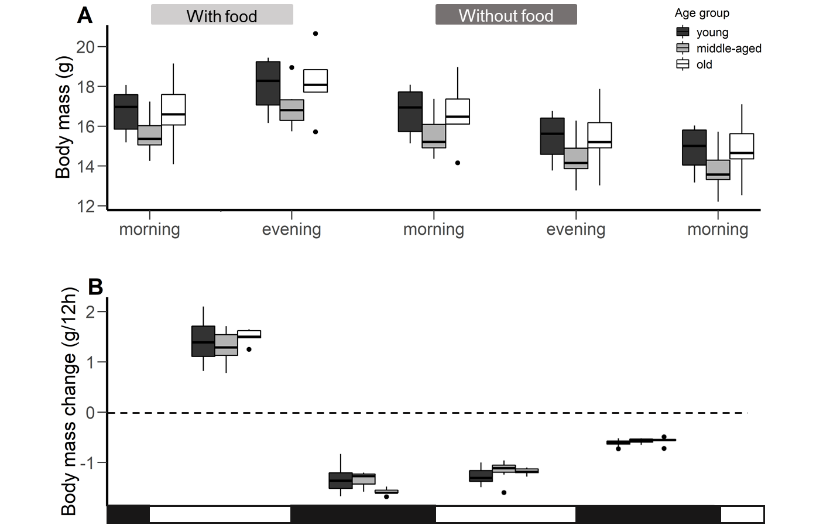


**Supplementary Figure 3| (A)** Body mass (g) measured during morning (just after lights on) and evening (just before lights off) of the three age groups during the days when birds have access to food and without food. **(B)** Body mass change over 12 hours during day and night when birds have access to food and without access to food. Boxplots show the median, the inter-quartiles calculated for each age group and the range between minimum and maximum values.

1. **Oxidative biomarkers and body temperature relationship**
   1. **Oxidative biomarkers and mean body temperature**

**
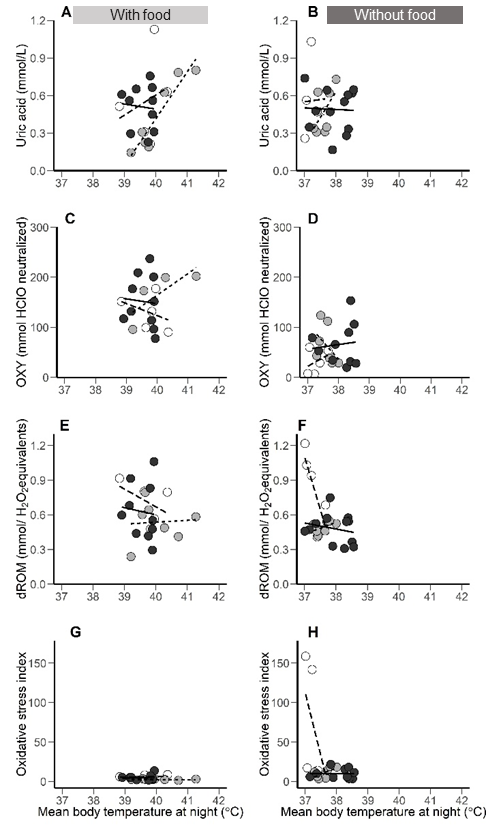
**

**Supplementary Figure 4|** The relationship between mean body temperature (°C) during the night with access to food (left panels) and without access to food (right panels). **(A** and **B)** Uric acid, **(C** and **D),** Non-enzymatic antioxidant capacity, **(E** and **F)** Oxidative damage, **(G** and **H)** Oxidative stress index in plasma concentration. Black filled circles and solid regression line represent young group, grey filled circles and dotted regression line the middle-aged, and white filled circles and the dashed regression line old group of birds.

- 1. **Oxidative biomarkers and energetic costs of rewarming**

**Estimation of energetic costs**

We used the formula (3) by McKechnie and Wolf (2004), a bioenergetic model, to estimate energetic costs. To quantify resting metabolic rate during rest-phase hypothermia we used the formula by Heldmaier and Ruf, (1992).

$E_{rewarm}=s \left( {Tb}_{norm}-{Tb}_{hyp} \right) M_{b}+D_{rewarm} ({RMR}_{hyp}+ \frac{BMR-{RMR}_{hyp}}{2} )$ (1)

$Q_{10}={\frac{{RMR}_{hyp}}{BMR}}^{\left( \frac{10}{{Tb}_{hyp}-{Tb}_{norm}} \right)}$ (2)

$D_{\mathrm{rewarm}}=\frac{\left( \mathrm{Tb}_{\mathrm{norm}}-\mathrm{Tb}_{\mathrm{hyp}} \right)}{\Delta t}$ (3)

Where

RMR_hyp_: Resting metabolic rate during hypothermia

BMR: Basal metabolic rate

Tb_hyp_: Body temperature during rest-phase hypothermia

Tb_norm_: Body temperature during resting, normothermic value

Δt: The difference between the two different time points, time point of normothelic level and time point of the hypothermic level of body temperature

**Basal metabolic rate measurements**

We quantified basal metabolic rate (BMR) for all birds as a rates of O_2_ consumption and CO_2_ production using an 8-channel open-flow positive-pressure respirometric system. BMR measurement was performed in individual chambers (n=8) inside a dark climate chamber with controlled temperature of 30 °C, which is within the thermoneutral zone of zebra finches (Calder, 1964). Individual chambers were built from typical commercial glass containers of 950 ml volume. Inside, we placed a tight metal cage to restrain birds from movements during the measurements. An inlet tube is inserted inside the chamber around 2cm above the bottom and outlet at the top which allowed a good air flow inside the chamber. The bottom of container was filled with 50g of white mineral oil (AnVit, Poland) to collect faeces. A fresh sample of air in standard pressure and room temperature was dried with silica gel driers and divided into 9 streams; eight was pumped into chambers with eight mass flow system pumps 2LPM (Sable System International, USA), and one for baseline reference. Air flow was set at 450ml min^-1^ through chambers and regulated separately for each chamber. Samples from chambers were regulated sequentially through Intelligent Multiplexer MUX (Sable System International, USA). Sub-sample of air stream was first analysed for water content and then dried with silica gel and magnesium perchlorate (Anhydrone, J.T. Baker, USA) columns before passing the CO_2_ and O_2_ analyser. Sample was analysed by Field Metabolic System FMS (Sable System International, USA). Representative O_2_ concentration value was calculated from the values recorded in the last 20s before switching channels similar to (Sadowska et al., 2015), when birds were in post absorptive state.

**
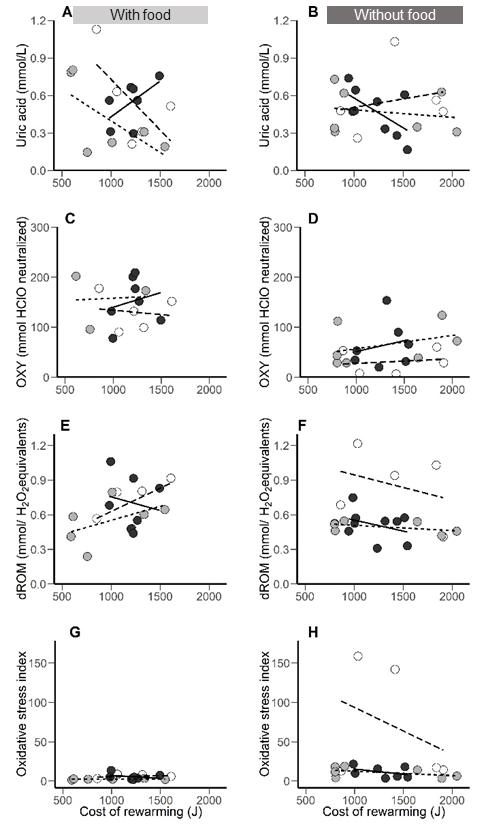
**

**Supplementary Figure 5|** The relationship between energetic costs during rewarming phase (Joules) between the night with access to food (left panels) and without access to food (right panels). **(A** and **B)** Uric acid, **(C** and **D)** OXY, a metric for non-enzymatic antioxidant capacity, **(E** and **F)** Oxidative damage, **(G** and **H)** oxidative stress index, calculated as the ratio of the oxidative damage to the total non-enzymatic antioxidant capacity in the plasma multiplied by 1000. Black filled circles and solid regression line represent young group, grey filled circles and dotted regression line the middle-aged, and white filled circles and the dashed regression line old group of birds.

References

Calder, W. A. (1964). Gaseous Metabolism and Water Relations of the Zebra Finch, Taeniopygia castanotis. *Physiol. Zool.* 37, 400–413. doi:10.1086/physzool.37.4.30152758.

Heldmaier, G., and Ruf, T. (1992). Body temperature and metabolic rate during natural hypothermia in endotherms*. J. Comp. Physiol. B* 162, 696–706.

McKechnie, A.E. and Wolf, B.O. (2004). The energetics of the rewarming phase of avian torpor. In Barnes, B.M. & Carey,C.M. (eds) Life in the Cold: Evolution, Mechanisms, Adaptation, and Application. Twelfth International Hibernation Symposium. Biological Papers of the University of Alaska 27, 265–274. Alaska, USA: Institute of Arctic Biology, University of Alaska Fairbanks.

**Supplementary Table 1**

Results of analysis of covariance (*ANCOVA*) of *T_b mean_*, *T_b min_*, *T_b max_* and *T_b median_* to test for the effect of age and day-night phase. The analysis was performed for three days that birds were undisturbed with access to food and thermoneutral conditions. The age factor (n_young_ = 12, n_middle-aged_ = 7 and n_old_ = 5) was included as a categorical variable. The day-night phase was set as a categorical variable. Body mass (g) was recorded just after lights on and was included as a covariate in the model. Statistics; F-value, degrees of freedom for numerator and denominator (df) and p value based on Satterthwaite’s method approximation. Bold letters indicate statistical significance of the effect smaller or equals to 0.05.

| Variable |  | Age group | | |  | Day-night phase | | |  | Body mass | | |  | Age group x  Day-night phase | | |
| --- | --- | --- | --- | --- | --- | --- | --- | --- | --- | --- | --- | --- | --- | --- | --- | --- |
|  |  | *F* | *df* | *p* |  | *F* | *df* | *p* |  | *F* | *df* | *p* |  | *F* | *df* | *p* |
| T_b_ mean |  | 0.78 | 2, 20 | 0.47 |  | 1935.6 | 1, 117 | **<0.001** |  | 1.28 | 1, 19.99 | 0.26 |  | 0.13 | 2, 117 | 0.87 |
| T_b_ min |  | 0.79 | 2, 20 | 0.46 |  | 2326.5 | 1, 117 | **<0.001** |  | 0.83 | 1, 20 | 0.37 |  | 0.16 | 2, 117 | 0.84 |
| T_b_ max |  | 2.83 | 2, 20 | 0.08 |  | 189.98 | 1, 20 | **<0.001** |  | 3.73 | 1, 20 | 0.06 |  | 0.44 | 2, 117 | 0.64 |
| T_b_ median |  | 0.48 | 2, 20 | 0.62 |  | 1581.69 | 1, 117 | **<0.001** |  | 0.96 | 1, 20 | 0.33 |  | 0.63 | 2, 117 | 0.53 |

**Supplementary Table 2**
Results of analysis of covariance (*ANCOVA*) of T_b mean_, T_b min_, T_b max_ and T_b median_ (°C) with access to food separately for day and night. Age group (n_young_ = 12, n_middle-aged_ = 7 and n_old_ = 5) was included as a categorical variable. Body mass (g) was recorded just after lights on and was included as a covariate in the model. Least squares means (LSM), standard error (SE), Statistics; F-value, degrees of freedom for numerator and denominator (df) and p value based on Satterthwaite’s method approximation. Bold letters indicate statistical significance of the effect smaller or equals to 0.05.

| Variable | |  | Young | |  | Middle-aged | |  | Old | |  | Age Group | | | | |  | Body mass | | | | |
| --- | --- | --- | --- | --- | --- | --- | --- | --- | --- | --- | --- | --- | --- | --- | --- | --- | --- | --- | --- | --- | --- | --- |
|  |  |  | LSM | SE |  | LSM | SE |  | LSM | SE |  | *F* |  | *df* |  | *p* |  | *F* |  | *df* |  | *p* |
| Day | T_b_ _mean_ |  | 41.8 | 0.1 |  | 41.7 | 0.1 |  | 41.7 | 0.1 |  | 0.35 |  | 2,20 |  | 0.71 |  | 1.07 |  | 2.20 |  | 0.31 |
|  | T_b min_ |  | 41.2 | 0.1 |  | 41.0 | 0.1 |  | 41.1 | 0.1 |  | 0.14 |  | 2,20 |  | 0.87 |  | 0.85 |  | 2,20 |  | 0.37 |
|  | T_b_ max |  | 42.8 | 0.0 |  | 42.5 | 0.1 |  | 42.5 | 0.1 |  | 2.06 |  | 2,20 |  | 0.15 |  | 2.91 |  | 2,20 |  | 0.10 |
|  | T_b_ median |  | 41.8 | 0.1 |  | 41.6 | 0.1 |  | 41.6 | 0.1 |  | 0.50 |  | 2,20 |  | 0.62 |  | 1.23 |  | 2,20 |  | 0.28 |
| Night | T_b_ mean |  | 39.8 | 0.1 |  | 39.8 | 0.1 |  | 39.7 | 0.2 |  | 0.15 |  | 2,20 |  | 0.86 |  | 0.03 |  | 2,20 |  | 0.85 |
|  | T_b_ min |  | 39.0 | 0.1 |  | 38.9 | 0.2 |  | 38.8 | 0.2 |  | 0.35 |  | 2,20 |  | 0.71 |  | 0.02 |  | 2,20 |  | 0.87 |
|  | T_b_ max |  | 41.5 | 0.1 |  | 41.3 | 0.2 |  | 41.2 | 0.2 |  | 1.14 |  | 2,20 |  | 0.34 |  | 4.78 |  | 2,20 |  | **0.04** |
|  | T_b_ median |  | 39.7 | 0.1 |  | 39.7 | 0.2 |  | 39.7 | 0.2 |  | 0.00 |  | 2,20 |  | 0.99 |  | 0.01 |  | 2,20 |  | 0.91 |

**Supplementary Table 3**

Results of linear mixed effect model analysis of uric acid, antioxidant capacity oxidative damage, and oxidative stress index (after log-transformation). Statistical model includes age group, food manipulation (with access to food and without access to food conditions) and the interaction as fixed factors. Initial body mass (g) as a covariate and individual identification was included as a random effect. Statistics; F-value, numerator, denominator of degrees of freedom (df) and p value based on Satterthwaite’s method approximation. Bold letters indicate statistical significance of the effect smaller or equals to 0.05.

| Variable |  | Age group | | |  | Food manipulation | | |  | Body mass | | |  | Age group x  Food manipulation | | |
| --- | --- | --- | --- | --- | --- | --- | --- | --- | --- | --- | --- | --- | --- | --- | --- | --- |
|  |  | *F* | *df* | *p* |  | *F* | *df* | *p* |  | *F* | *df* | *p* |  | *F* | *df* | *p* |
| Uric acid |  | 1.18 | 2, 19.82 | 0.32 |  | 0.00 | 1, 19.69 | 0.95 |  | 1.43 | 1, 19.71 | 0.25 |  | 0.00 | 2, 19.76 | 0.99 |
| Antioxidant capacity |  | 1.27 | 2, 39 | 0.29 |  | 38.13 | 1, 39 | **<0.001** |  | 1.37 | 1, 39 | 0.25 |  | 1.75 | 2, 39 | 0.19 |
| Oxidative damage |  | 5.79 | 2, 21.84 | **0.01** |  | 0.20 | 1, 21.25 | 0.66 |  | 1.80 | 1, 20.66 | 0.19 |  | 2.59 | 2, 21.58 | 0.09 |
| Log(Oxidative stress index) |  | 6.18 | 2, 37 | **0.004** |  | 38.05 | 1, 37 | **<0.001** |  | 2.51 | 1, 20.63 | 0.12 |  | 2.74 | 2, 37 | 0.07 |

**R script for the final analysis presented in the manuscript and supplementary material**

if (FALSE) {

install.packages("glmm")

install.packages("data.table")

install.packages("glmmTMB")

install.packages("car")

install.packages("dplyr")

install.packages("lsmeans")

install.packages("lmerTest")

}

# load packages

library(lme4)

library (car)

library(dplyr)

library(lmerTest)

library(tidyverse)

library(emmeans)

# read file

tb_stats <- read.csv("data_analysis_final_manuscript.csv", sep = ",", header = T)

summary(tb_stats)

View(tb_stats)

# age.class, age group (young, middle-aged, young)

# daynight, photoperiod cycle of day and night

# bm, body mass measured just lights on

# ID, individual identification number

# mean.bt, mean body temperature

# min.bt, minimum body temperature

# median.bt, median body temperature

# relevel function, changes the order of the age group with the young group to appear first

tb_stats$age.class <- relevel(as.factor(tb_stats$age.class), ref="y")

**# Does mean body temp during the night differ between the experimental days?**

lm_tb <- lmer(mean.bt ~ age.class * daynight + bm + (1|ID),

data=tb_stats %>%

filter(food.manip==c("with")) %>%

filter(exp.day %in% c("day9", "day5", "day8")))

summary(lm_tb)

anova(lm_tb)

Anova(lm_tb)

plot(lm_tb)

plot(residuals(lm_tb), id=0.05, idLabels= ~.obs)

plot(lm_tb,id=0.05,idLabels=~.obs) # via lme4 package

qqnorm(residuals(lm_tb))

**# Does night-time body temperature differ between age groups during regular conditions with access to food ?**

# Analysis of covariance

# age as a categorical factor

lm_tb_mean_nig_with<- lm(mean.bt ~ age.class + bm,

data=tb_stats %>%

filter(food.manip==c("with")) %>%

filter(daynight == c("night")) %>%

filter(exp.day==c("day9")))

summary(lm_tb_mean_nig_with)

anova(lm_tb_mean_nig_with)

plot(lm_tb_mean_nig_with)

**# Does night-time body temperature differ between age groups in response to a day-long food deprivation?**

lm_tb_nig_without <- lm(mean.bt ~ age.class + bm,

data=tb_stats %>%

filter(food.manip==c("without3")) %>%

filter(daynight==c("night")))

summary(lm_tb_nig_without)

anova(lm_tb_nig_without)

par(mfrow=c(2,2))

plot(lm_tb_nig_without)

plot(residuals(lm_tb_nig_without), id=0.05, idLabels= ~.obs)

plot(lm_tb_nig_without,id=0.05,idLabels=~.obs) # via lme4 package

qqnorm(residuals(lm_tb_nig_without))

**# Similar analysis for age as a continuous variable**

**# table s2**

lm_tb_nig_with2 <- lm(mean.bt ~ yr.old + bm, data=tb_stats %>%

filter(food.manip==c("with")) %>%

filter(exp.day == c("day9")) %>%

filter(daynight == c("night")))

summary(lm_tb_nig_with2)

anova(lm_tb_nig_with2)

plot(lm_tb_nig_with2)

par(mfrow=c(2,2))

plot(lm_tb_nig_with2)

plot(residuals(lm_tb_nig_with2), id=0.05, idLabels= ~.obs)

plot(lm_tb_nig_with2,id=0.05,idLabels=~.obs) # via lme4 package

qqnorm(residuals(lm_tb_nig_with2))

**# table s3**

lm_tb_nig_without2 <- lm(mean.bt ~ yr.old + bm, data=tb_stats %>%

filter(food.manip==c("without3")) %>%

filter(daynight==c("night")))

summary(lm_tb_nig_without2)

anova(lm_tb_nig_without2)

par(mfrow=c(2,2))

plot(lm_tb_nig_without2)

plot(residuals(lm_tb_nig_without2), id=0.05, idLabels= ~.obs)

plot(lm_tb_nig_without2,id=0.05,idLabels=~.obs) # via lme4 package

qqnorm(residuals(lm_tb_nig_without2))

**# Linear mixed effect analysis with age group, food manipulation and the interaction as fixed effects**

**# figure2**

lm_tb_mean_nig2 <-

lmer(mean.bt ~ age.class * food.manip + bm + (1|ID),

data=tb_stats %>%

filter(exp.day %in% c("day10","day9", "day8", "day5")) %>%

filter(daynight==c("night")))

summary(lm_tb_mean_nig2)

anova(lm_tb_mean_nig2)

plot(lm_tb_mean_nig2)

plot(residuals(lm_tb_mean_nig2), id=0.05, idLabels= ~.obs)

plot(lm_tb_mean_nig2,id=0.05,idLabels=~.obs) # via lme4 package

qqnorm(residuals(lm_tb_mean_nig2))

summary(emmeans(lm_tb_mean_nig2,specs = c("age.class", "food.manip")))

summary(emmeans(lm_tb_mean_nig2,specs = c("food.manip")))

print(emmeans(lm_tb_mean_nig2, pairwise ~ age.class|food.manip),adjust="Tukey")

**# Oxidative stress biomarkers**

**# uric acid**

lm_ua1 <- lmer(UA ~ age.class * exp.day + bm + (1|ID), data= tb_stats %>%

filter(exp.day %in% c("day10", "day16")) %>%

filter(daynight == "night"))

summary(lm_ua1)

anova(lm_ua1)

plot(lm_ua1)

**# antioxidant capacity**

lm_oxy1 <- lmer(oxy ~ age.class * exp.day + bm + (1|ID), data= tb_stats %>%

filter(exp.day %in% c("day10", "day16")) %>%

filter(daynight == "night"))

summary(lm_oxy1)

anova(lm_oxy1)

plot(lm_oxy1)

**# oxidative damage**

lm_od1 <- lmer(drom ~ age.class * exp.day + bm. + (1|ID),

data= tb_stats %>%

filter(exp.day %in% c("day10", "day16")) %>%

filter(daynight == "night"))

summary(lm_od1)

anova(lm_od1)

plot(lm_od1)

**# oxidative stress index**

lm_os1 <- lmer((os.index*1000) ~ age.class * exp.day + bm + (1|ID),

data= tb_stats %>%

filter(exp.day %in% c("day10", "day16")) %>%

filter(daynight == "night"))

summary(lm_os1)

anova(lm_os1)

plot(lm_os1)
